# Relationship Between Frequency of Physical Activity, Functional Mobility, and Self-Perceived Health in People with Different Levels of Pain: A Cross-Sectional Study

**DOI:** 10.3390/jfmk9040198

**Published:** 2024-10-21

**Authors:** Ángel Denche-Zamorano, Diana Salas-Gómez, Sabina Barrios-Fernandez, Pablo Tomás-Carus, José Carmelo Adsuar, Jose A. Parraca

**Affiliations:** 1 Promoting a Healthy Society Research Group (PHeSO), Faculty of Sports Sciences, University of Extremadura, 10003 Cáceres, Spain; denchezamorano@unex.es (Á.D.-Z.); jadssal@unex.es (J.C.A.); 2 Departamento de Desporto e Saúde, Escola de Saúde e Desenvolvimento Humano, Universidade de Évora, 7004-516 Evora, Portugal; ptc@uevora.pt (P.T.-C.); jparraca@uevora.pt (J.A.P.); 3 Social Impact and Innovation in Health (InHEALTH), Faculty of Sports Sciences, University of Extremadura, 10003 Cáceres, Spain; 4 Comprehensive Health Research Centre (CHRC), Universidade de Évora, 7004-516 Evora, Portugal; 5 Interdisciplinary Centre for the Study of Human Performance (CIPER), Faculty of Human Kinetics, University of Lisbon, 1649-004 Lisbon, Portugal

**Keywords:** exercise, health, walking, steps, pain, health care

## Abstract

**Background**: Habits including regular physical activity are necessary for maintaining good health. Functional mobility, including walking and going up and down stairs, is essential for personal autonomy and well-being. Pain is a condition related to biological and psychosocial aspects that influence people’s lives. **Objective**: The main objectives of this study were (1) to analyse the associations between physical activity frequency (PAF) and self-perceived health (SPH) and functional mobility (walking and going up and down stairs) in middle-aged and older people living in Spain with different pain levels; and (2) to analyse the risk factors for having a negative SPH and functional mobility difficulties by calculating the probabilistic risks adjusted by different variables (sex, body mass index, social class, civil status, smoking status, pain level, and PAF). **Methods**: A cross-sectional study based on the European Health Survey data in Spain (EHSS 2014-2020) and The Spanish National Health Survey (SNHS 2017) was carried out, with a final sample of 21,152 participants with ages between 40 and 79 years. **Results**: Associations between high pain levels and worse SPH and difficulties in walking and climbing stairs were found. Lower PAF levels were associated with higher-probability risks of having a negative SPH and difficulties in walking and climbing stairs. **Conclusions**: Physical inactivity emerged as an important risk factor for worse SPH and functional mobility. These associations underline the importance that PA programmes can play in the improvement of health and functional mobility, as well as in other aspects, in people with pain.

## 1. Introduction

Physical activity (PA) is any bodily movement produced by skeletal muscles that requires energy expenditure and encompasses movements performed during activities of daily living, at work or during leisure time [1,2]. PA has multiple physical and mental health benefits but should be undertaken based on evidence-based recommendations regarding frequency, intensity, and duration. Therefore, the World Health Organisation updated its guidelines on PA recommendations and sedentary behaviour for different age groups and conditions [3]. Thus, in adults (18–64 years), a minimum of 150 to 300 min of moderate-intensity aerobic PA, or 75 to 150 min of vigorous-intensity aerobic PA, or an equivalent combination of both, plus strength work and limiting the time spent doing sedentary activities as much as possible, is recommended. In older adults (over 65 years), it is also recommended to introduce multicomponent workouts in combination with the above, prioritising balance and strength to improve functional capacity and prevent falls [3]. Regarding the recommended physical activity for people with pain, it has been shown that a hypoalgesic effect occurs with both aerobic and strengthening exercises [4,5]. Performing aerobic exercise at a low to moderate intensity of 50–60% of the maximum heart rate has an analgesic effect in chronic pain pathology [4,6]. On the other hand, strengthening exercises are effective in chronic pain and are well tolerated [4]. Regarding the frequency of physical activity, there is no solid evidence to recommend specific parameters in terms of frequency for people with chronic pain [4].

Regarding strengthening exercises, isometric contractions with loads of 10–30% are sufficient to achieve a hypoalgesic effect, especially when prolonged until failure. A 3-min wall squat programme is also effective. In terms of intensity, vigorous aerobic exercise (70% HRR, three sessions of 25 min) has a greater hypoalgesic effect than moderate exercise (50% HRR) [7]. However, other studies suggest that aerobic exercise at a low (40% HRR) or moderate (55% HRR) intensity produces a greater primary and delayed hypoalgesic response than intense exercise (70% HRR) [8]. Some studies find no differences in hypoalgesia based on the intensity of aerobic exercise [4,9].

Compliance with these recommendations is vital since, in addition to influencing functionality, health, wellness, and quality of life in adults and older people with pain [10,11], the consequences of physical inactivity and sedentary lifestyles significantly impact health systems [12,13,14]. In this vein, the evidence, although still scant, suggests that physical activity may reduce pain and improve function in some adults with chronic pain, while presenting few adverse events [15].

The World Health Organisation’s (WHO) International Classification of Functioning, Disability, and Health (ICF) places mobility within the Activities and Participation component in Chapter 4. Mobility is defined broadly and includes aspects such as changing one’s body position or location or walking, running, or climbing. Within this chapter, there are different categories including walking and moving (d450–d469) [16]. According to the American Occupational Therapy Association (AOTA), functional mobility includes, among others, moving from one position or place to another, and functional ambulation. This functional mobility is included in the Activities of Daily Living (ADLs), which are those activities oriented to the care of one’s own body and which are routinely completed [17]. Thus, both organisations and scientific evidence highlight the importance of individuals becoming or remaining functional concerning mobility to achieve independence, well-being, and health. PA programmed can help to prevent decline [18] or improve physical function and mobility [19,20].

Self-perceived health (SPH) is a summarised declaration on how multiple aspects of health, both subjective and objective, are combined in an individual’s perception of their health and is considered an important indicator of health status [21]. SPH is considered a reliable predictor of health status by integrating objective knowledge of potential conditions with the individual’s interpretation of physical and mental signs [22]. Moreover, it is also considered an indicator of lifestyle-related health status, as it is associated with changes in weight, smoking, exercise, and rest [23]. Thus, functional mobility conditions, among other factors such as body mass index, chronic diseases, medications, and muscle strength issues, have a strong effect on SPH [24]. Pain’s newest definition reads that it is an unpleasant sensory and emotional experience associated with actual or potential tissue damage, influenced by vital experiences and biological, psychological, and social factors. People who experience pain may have adverse effects at psychological and social levels, impacting their well-being, although, in principle, its function should be adaptive [25]. The intensity of pain or some kinds of pain, such as chronic pain, are associated with worse SPH [26].

Several previous studies have examined the relationship between the frequency of physical activity and self-perceived health across various populations. These populations include the general adult population, adults with diabetes, and older adults, for example [27,28,29]. The findings indicate that individuals who engaged in physical activity several times a week had a lower prevalence of poor self-perceived health. Conversely, the risk of negative self-perception was higher among inactive individuals [27,28]. Similarly, in older adults, better self-perceived health was associated with higher levels of physical activity [29]. On the other hand, physical activity is important for all adults, including those with functional limitations, as it can prevent or delay age-related declines in functioning [30].

However, to the best of the authors’ knowledge, no studies have evaluated the relationship between physical activity frequency and self-perceived health in populations with varying degrees of pain, nor the association between physical activity frequency and functional mobility. Therefore, the main objectives of this cross-sectional study were: (1) to analyse the associations between PAF and SPH, 500 m walking difficulties without assistance, and walking up or down 12 or more steps in middle-aged and older people living in Spain with Low, Medium, and High Pain, while also comparing negative SPH prevalence and difficulties in walking and going up or down stairs as a function of PAF; (2) to analyse the risk factors for having a negative SPH, walking difficulties without assistance, and walking up or down 12 or more steps, by calculating the probabilistic risks adjusted by age, sex, BMI (body mass index), social class, civil status, smoking status, pain level, and PAF.

This study hypothesised that: (1) PAF will be associated with SPH and difficulties walking and walking up or down stairs; people with lower PAF will have higher negative SPH proportions and difficulties in walking and walking up or down stairs; (2) people who do not engage in PA or perform low frequencies of PA will have higher probability risks of having negative SPH, as well as walking difficulties without assistance and walking up or down 12 or more steps.

## 2. Materials and Methods

### 2.1. Study Design

This cross-sectional study was based on data published by the Spanish Ministry of Health (SMH) and the Spanish National Institute of Statistics (SNIS) corresponding to The European Health Surveys in Spain 2014 and 2020 (EHSS 2014 and EHSS 2020) and the Spanish National Health Survey 2017 (SNHS 2017). The data are publicly available, free to use and previously anonymised. As these are anonymised public data, they are considered non-confidential data and their use does not require following data protection principles, the need for informed consent documents, or the approval of an accredited ethics committee, based on Regulation 2016/679 of the European Parliament and of the Council of 27 April 2016 on the protection of natural persons concerning the processing of personal data and on the free movement of personal data, and derogating from Directive 95/46/EC, these data are anonymous, public, and are considered non-confidential data.

### 2.2. Data Resources

#### 2.2.1. European Health Surveys in Spain 2014 and 2020 (EHSS)

The EHSS is the Spanish part of the European Health Interview Survey (EHIS), a survey coordinated by the European Statistical Office (Eurostat) and conducted in all member countries of the European Union (EU). The EHIS aim is to measure health status, health determinants (related to lifestyle, such as sedentary lifestyles) and the use of health care services by (and access limitations for) EU citizens, in a harmonised way and with a high degree of comparability between Member States (MSs). The EHIS is coordinated by the European Statistical Office (Eurostat) and carried out by those responsible for each MS. In the case of Spain, it was performed by the National Statistics Institute (NSI) in collaboration with the SMH. The questionnaire consists of four modules: health status, health determinants, health care, and contextual (socio-demographic) variables. In Spain, INE-trained and accredited interviewers administered the questionnaire, in the case of the EHSS 2014 between January 2014 and January 2015, and in the EHSS 2020, between July 2019 and July 2020.

A three-stage stratified random sampling system (random choice of a municipality within the strata, random choice of a dwelling within the selected municipality, and random choice of an adult from the selected dwelling) was used to select participants in both surveys. Participants were informed of their choice and participated voluntarily in the survey, and accepted the processing and anonymous disclosure of their data. If accepted to participate, the interviewers conducted the survey by making an appointment with the participant. All data concerning sample calculations, data processing, treatment of missing data, sample selection, and other methodological details of the surveys were extensively described in their methodologies of the European Health Survey in Spain. The raw data for both surveys are available at EHSS Data.

#### 2.2.2. The Spanish National Health Survey 2017 (SNHS)

The SNHS is a series of surveys, initiated in 1987 and carried out periodically, and is the most important information source on perceived health in the resident population in Spain. As well as in the SNHS, the aim is to provide information on the population’s health status, in addition to other health determinants and the access to and use of health services. In fact, since 2009, both surveys, the EHSS and the SNHS, alternate every two and a half years, sharing a large number of variables. Commissioned by the Spanish Ministry of Health, it is carried out by the NSI, which is responsible for selecting participants, training interviewers, and processing and publishing the data. The last SNHS conducted in Spain was the SNHS 2017, where trained and accredited interviewers conducted the survey (adult questionnaire) between October 2016 and October 2017.

Participant selection was carried out using stratified tri-stage sampling similar to that described in the EHSS. Municipalities were stratified, municipalities were randomly selected, households were randomly selected from those municipalities, and finally, one adult was randomly selected from each household. The selected participants were contacted and proposed to participate voluntarily and to be able to disclose their data anonymously. If so, the interviewers made an appointment with the participants to conduct the survey. All methodological details of the survey were published in the SNHS 2017 methodology Spanish National Health Survey, and the anonymized microdata were published on SNHS Data.

### 2.3. Participants

**Figure 1:**
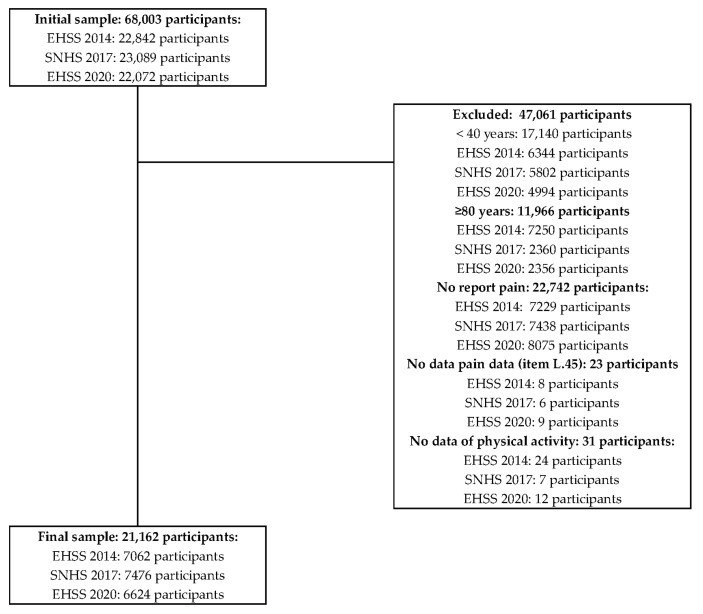
Sample flow chart.

The initial sample consisted of 68,003 adults aged 15 years and older living in Spain who participated in the EHSS 2014 (22,842 participants), the SNHS 2017 (23,089 participants), and the EHSS 2020 (22,072 participants) surveys. The target population for this study was middle-aged and older people with pain who participated in the aforementioned surveys and submitted data on PAF. Therefore, the inclusion criteria were (1) having participated in the EHSS 2014, SNHS 2017, or EHSS 2020; (2) being aged between 40 and 79 years; (3) living with pain (item Q.45 in all three surveys); and (4) submitting data about PAF (item Q.112 in all three surveys). Exclusion criteria were (1) being younger than 40 years or the same or older than 80 years; (2) not presenting pain; or (3) no PAF data. Figure 1 shows the flow chart from an initial sample to the final one composed of 21,162 participants.

### 2.4. Variables

#### 2.4.1. Outcome Variables

*Self-Perceived Health (SPH)* was extracted from the data provided by item Q.21 on the three surveys. This item asks the participants about their health state over the last twelve months, with the following response options: “Very good”, “Good”, “Fair”, “Bad”, or “Very bad”. For this study, participants were grouped according to the answers given as follows: negative SPH (Fair, Poor and Very Poor) and Positive SPH (Good and Very Good).

*500 m Walking Difficulties (500mWD)* was taken from item Q.37 in which participants were asked about how difficult it was for them to walk 500 m on level ground without assistance, with the following response options: “No, no difficulty”; “Yes, some difficulty”; “Yes, a lot of difficulty”; “I can’t do it at all”; or don’t know or don’t answer (DK/DA). For this study, we considered No (participants who answered “No, no difficulty”) and Yes (participants who answered “Yes, some difficulty” or “Yes, a lot of difficulty”). This variable did not include participants who answered “I can’t do it at all”, as this level of response included wheelchair users and amputees, among others.

*12 Steps Difficulties (12SD)* was taken from item Q.38 in which participants were asked about the difficulty of walking up or down 12 steps, with the following response options: “No, no difficulty”; “Yes, some difficulty”; “Yes, a lot of difficulty”; “I can’t do it at all”; or (DK/DA). For this study, we simply considered No (participants who answered “No, no difficulty”) and Yes (participants who answered “Yes, some difficulty” or “Yes, a lot of difficulty”). As with the previous variable, this variable did not include participants who responded “I can’t do it at all”.

#### 2.4.2. Socio-Demographic Variables

*Age* was extracted from the AGEa variable, collecting the participants’ age in years.

*Sex* was extracted from the SEXOa variable, grouping participants into men and women.

*Body Mass Index (BMI)* was extracted from the BMIa variable, which grouped participants according to their BMI (kg/m^2^) into the following options: “underweight” (<18.5), “normal” (≥18.5 and <25), “overweight (≥25 and <30)”, and “obesity” (≥30). Participants who did not submit data on this variable were not included in the analyses with this variable.

*Social Class* was extracted from the “CLASEPR” variable, which grouped participants according to their social class, presenting six possible levels: “I”, “II”, “III”, “IV”, “V”, and “VI” [31]. The construction of this variable was detailed in the survey methodology and previous articles. Participants who did not submit data on this variable were not included in the analyses with this variable.

*Civil Status* was extracted from the Q.4b variable. Participants were grouped according to their marital status: “Single”, “Married”, “Divorced”, “Legally separated”, and “Widowed”. Participants who did not submit data on this variable were not included in the analyses with this variable.

*Smoking* was extracted from answers given to item Q.121 with the following options: “Yes, I smoke daily”; “Yes, but I do not smoke daily”; “I do not currently smoke but I have smoked”; and “I do not smoke and have never smoked regularly”. For this study, they were considered as Smokers, Occasionally, Ex-Smokers, and Non-Smokers. Participants who did not submit data on this variable were not included in the analyses with this variable.

*Physical Activity Frequency (PAF)* was drawn from item Q.112. This item asked participants about their PAF in their leisure time. Possible answers were “I do not exercise. I spend my free time almost completely sedentary (reading, watching TV, going to the cinema, etc.)”; “I do some occasional physical activity or sport (walking or cycling, gardening, gentle gymnastics, recreational activities requiring light effort, etc.)”; “I do physical activity several times a month (sports, gymnastics, running, swimming, cycling, etc.)”; “I do physical activity several times a month (sports, gymnastics, running, swimming, cycling, etc.), team games, etc.)”; and “I do sports or physical training several times a week”. For this study, they were considered as Never, Occasionally, Frequently, and Very Frequently.

*Pain Level* was extracted from variable Q.45. Participants were grouped according to the degree of pain experienced in the last 4 weeks, with possible response options being “None”, “Very mild”, “Mild”, “Moderate”, “Severe”, “Extreme”, and “DK/DA”. As indicated in the inclusion and exclusion criteria, participants who answered none or DK/DA were excluded. Thus, for this study, the remaining participants were grouped into these variable groups: Low Pain (participants with “Very mild” or “Mild” pain), Medium Pain (participants with “Moderate” pain), and High Pain (participants with “Severe” or “Extreme” pain).

### 2.5. Statistical Analysis

The data distribution for the continuous variable Age was checked using the Kolmogorov–Smirnov test. A descriptive analysis in which the median age, the Interquartile Range, and the absolute and relative frequencies presented by the participants in all categorical variables and according to their Pain Level was performed. The Kruskal–Wallis’s test was used to check differences in participants’ age by Pain Level. Associations between the Pain Level and all categorical variables in the study were analysed using the Chi-square test. The same tests were also used to analyse the associations between PAF and SPH, the 500mWD, and the 12SD. The post hoc pairwise z-test for independent proportions was included to compare the proportions presented by the different Pain Level groups on the categorical variables. Also, a post hoc pairwise z-test for independent proportions was used to find possible differences in proportions on these variables as a function of PAF in people with Low, Medium, and High Pain. In all cases, the strength of these associations was assessed by calculating Cramer’s Phi and V coefficients.

Finally, multiple binary logistic regression models were performed to analyse the risk factors for negative SPH, presenting 500mWD or 12SD, respectively, taking as independent variables Age, Sex, BMI, Civil Status, Social Class, Smoking Status, Pain Level, and PAF. For multiple logistic binary regressions, the assumptions of the absence of influential factors, independence, and no collinearity were tested. All analyses were performed with the IBM SPSS Statistics for Windows, Version 25.0. Armonk, NY, USA: IBM Corp software, establishing a minimum level of significance of less than 0.05.

## 3. Results

### 3.1. Descriptive Analysis of the Sample

The Kolmogorov–Smirnov test found insufficient evidence to assume that data for the Age variable followed a normal distribution (*p* < 0.001). The descriptive analysis of the sample, as well as the associations found, the strength of these associations, and the differences in proportions between Pain Level groups, are shown in Table S1. The median age of the sample was 60 years, being higher in the High Pain group (61 years) than in the Low (58 years) and Medium ones (60 years), finding intergroup differences (*p* < 0.001). Pain Level was related to Sex (*p* < 0.001), as 61.4% of the sample were women, compared to 38.6% being men. The proportion of women was higher when the Pain Level was higher: 56.1% (Low Pain) vs. 64.4% (Medium Pain) vs. 70.0% (High Pain). Significant differences in proportions between the three groups were found (*p* < 0.05). Dependence relationships were also found between Pain Level and the other categorical variables: BMI (*p* < 0.001), Social Class (*p* < 0.001), Smoking Status (*p* = 0.033), PAF (*p* < 0.001), SPH (*p* < 0.001), 500mWD (*p* < 0.001), and 12SD (*p* < 0.001). The overweight prevalence was 41.0% and the obesity prevalence 23.3%; the higher the obesity prevalence, the higher the Pain Level: 20.4% (Low Pain) vs. 24.1% (Medium Pain) vs. 29.2% (High Pain). This showed significant differences between the three groups (*p* < 0.05).

Negative SPH prevalence followed the same line, going from 37.2% in people with Low Pain to 64.3% and 80.9% in people with Medium and High Pain (*p* < 0.05); 500mWD went from 10.2% (Low Pain) to 25.5% and 46. 6% in people with Medium and High Pain (*p* < 0.05); 12SD, from 15.5% (Low Pain) to 33.4% and 55.4% in people with Medium and High Pain, with *p* < 0.05 (Table S1). Conversely, people with higher PAF proportions were higher than those reporting lower Pain Levels. People with Low Pain proportions performing PA Frequently or Very Frequently were 9.0% and 10.5%, respectively, and 7.6% and 7.7% in people with Medium Pain and 5.8% and 5.9% in people with High Pain. Significant differences were found in all these proportions between Pain Level groups (*p* < 0.05).

### 3.2. Self-Perceived Health and Physical Activity Frequency

**Figure 2:**
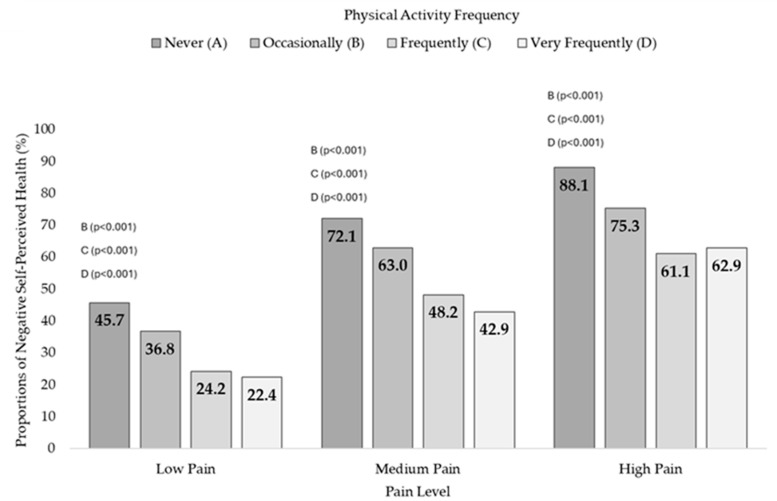
Negative Self-Perceived Health according to physical activity frequency in people with Low, Medium, and High Pain. The negative SPH prevalences in physically inactive people reached 45.7% in people with Low Pain, 72.1% in people with Medium Pain, and 88.1% in people with High Pain, being lower in people with higher PAF in each of the pain conditions. The letters indicate differences in proportions on Negative Self-Perceptive Health as a function of PAF in people with Low, Medium, and High Pain (*p*-value of post hoc pairwise z-test for independent proportions).

Dependency relationships between the PAF and the SPH in the three pain levels were found: Low Pain (X^2^ = 274.5, df = 3, *p* < 0.001, V = 0.164), Medium Pain (X^2^ = 251.1, df = 3, *p* < 0.001, V = 0.189), and High Pain (X^2^ = 204.6, df = 3, *p* < 0.001, V = 0.228). Moreover, it was found that people in the three pain groups who never performed PA had higher negative SPH prevalences, with significant differences with the rest of the groups (Table S2). Figure 2 shows the negative SPH prevalences according to the PAF for each pain condition.

### 3.3. 500-Metre Walking Difficulties and Physical Activity Frequency

**Figure 3:**
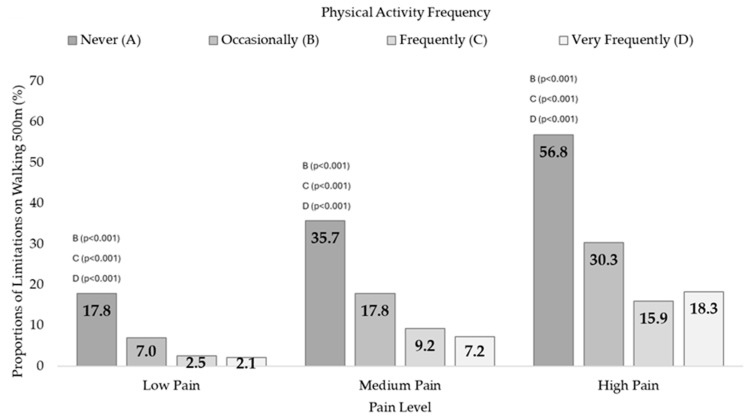
Walking 500 m difficulties according to physical activity frequency in people with Low, Medium, and High Pain. The higher prevalence was found in those who did not perform PA compared to the other PAF groups in the three pain conditions (Low, Medium, High). The letters indicate differences in proportions on Walking 500 m difficulties as a function of PAF in people with Low, Medium, and High Pain (*p*-value of post hoc pairwise z-test for independent proportions).

Dependence relationships were also found between PAF and 500mWD in the three pain levels: Low Pain (X^2^ = 424.0, df = 3, *p* < 0.001, V = 0.204), Medium Pain (X^2^ = 428.7, df = 3, *p* < 0.001, V = 0.250), and High Pain (X^2^ = 360.0, df = 3, *p* < 0.001, V = 0.314). Differences in proportions were found between those who never performed PA and the rest of the groups (Table S3). Figure 3 shows the difficulties in walking 500 m proportions as a function of PAF for every pain condition.

### 3.4. 12 Steps Difficulties and Physical Activity Frequency

**Figure 4:**
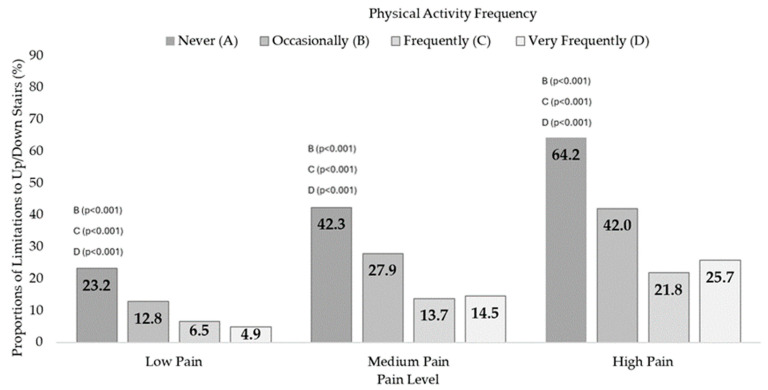
Up or down 12-step stairs difficulties according to the physical activity frequency in people with Low, Medium, and High Pain. The highest prevalences were found in those who never performed physical activity. The letters indicate differences in proportions on Up or down 12-step stairs difficulties as a function of PAF in people with Low, Medium, and High Pain (*p*-value of post hoc pairwise z-test for independent proportions).

Similarly, dependency relationships were found between PAF and 12SD in the three pain levels: Low Pain (X^2^ = 336.6, df = 3, *p* < 0.001, V = 0.182), Medium Pain (X^2^ = 324.7, df = 3, *p* < 0.001, V = 0.217), and High Pain (X^2^ = 308.6, df = 3, *p* < 0.001, V = 0.293). Differences in proportions were also found between those who never performed PA and the other groups (Table S4). Figure 4 shows the 12SD prevalences according to PAF in every pain condition.

### 3.5. Multiple Binary Logistic Regression

**Figure 5:**
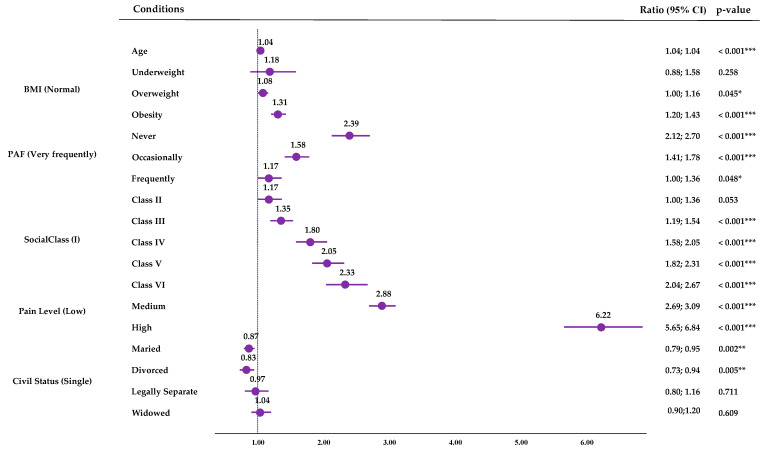
Multiple binary logistic regression for SPH. CI (Confidence Interval); BMI (body mass index); * (*p*-value < 0.05); ** (*p*-value < 0.01); *** (*p*-value < 0.001).

When obtaining the results of the binary multiple logistic regression for SPH, taking as reference the highest PAF (Very Frequently), lower PAF levels were associated with higher-probability risks of perceiving a negative SPH: Never (OR: 2.39; CI95%: 2.1; 2.70; *p* < 0.001), Occasionally (OR: 1.58; CI95%: 1.41; 1.78; *p* < 0.001), and Frequently (OR: 1.17; CI95%: 1.00; 1.36; *p* < 0.001). Along with lower PAF, advanced age, obesity or overweight, lower social class, higher pain levels, and some marital statuses were found to be associated with a higher risk of Negative SPH (Figure 5). The resulting model explained 26.0% of the variance (R^2^). Full details of the model are presented in Table S5.

**Figure 6:**
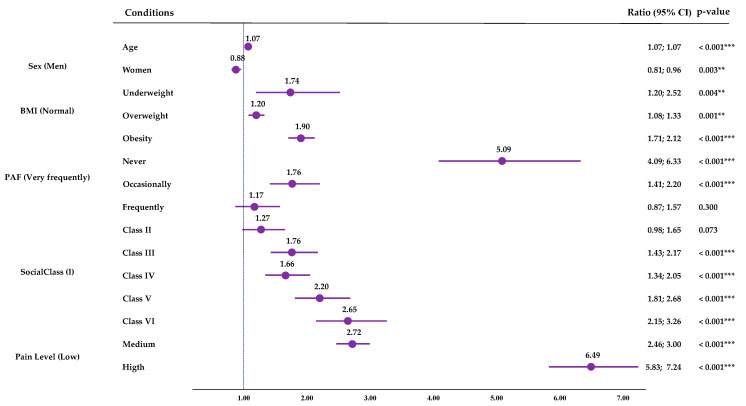
Multiple binary logistic regression for the 500 m walking difficulties. CI (Confidence Interval); BMI (body mass index); ** (*p*-value < 0.01); *** (*p*-value < 0.001).

Low PAF was also associated with higher odds of reporting 500mWD compared to the higher PAF group: Never (OR: 5.09; CI95%: 4.09; 6.33; *p* < 0.001) and Occasionally (OR:1.76; CI95%: 1.41; 2.20; *p* < 0.001). Older age, being male, belonging to a lower social class, and having higher pain levels were also found to increase the probability risk (Figure 6). Table S6 shows the model obtained, which explained 32.7% of the variance (R^2^).

**Figure 7:**
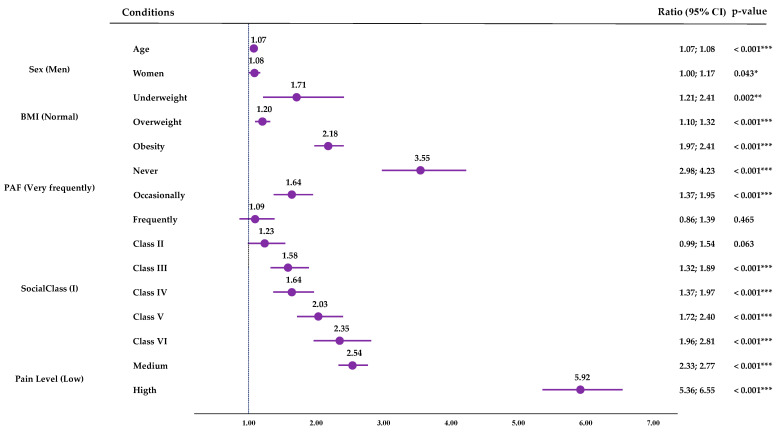
Multiple binary logistic regression for the 12-step difficulties. CI (Confidence Interval); BMI (body mass index); * (*p*-value < 0.05); ** (*p*-value < 0.01); *** (*p*-value < 0.001).

Table S7 shows the multiple binary logistic regression results for 12SD. The resulting model explained 32.9% of the variance (R^2^). In this model, low PAF was associated with an increased probability risk of reporting difficulties in walking up or down 12 steps: Never (OR: 3.55; CI95%: 2.98; 4.23; *p* < 0.001) and Occasionally (OR: 1.64; CI95%: 1.37; 1.95; *p* < 0.001). Along with low PAL levels, advanced age, being female, underweight, overweight or obesity, belonging to a lower social class and higher pain levels were associated with higher risks of having difficulties in walking up or down 12 steps (Figure 7).

## 4. Discussion

### 4.1. Main Findings

This study aimed: (1) to analyse the associations between PAF and SPH, 500 m walking difficulties without assistance, and walking up or down 12 or more steps in middle-aged and older people living in Spain with Low, Medium, and High Pain, while also comparing negative SPH prevalence and difficulties in walking and going up or down stairs according to PAF; (2) to analyse the risk factors for having a negative SPH, walking difficulties without assistance, and difficulties going up or down 12 or more steps, by estimating the probabilistic risks adjusted by age, sex, BMI, social class, civil status, smoking status, pain level, and PAF.

After performing the descriptive analysis to characterise the sample, it was observed that the higher the pain level, the higher the negative SPH prevalence. The same applies to the difficulties in walking 500 m (500WD) or going up or down stairs (12SD), all showing significant differences. There are also higher proportions of women with pain in all groups, so the gender perspective should be considered when addressing this challenge. This fact is supported by studies in the United States, where more adult women are categorised as having localised chronic pain (women, 56.4%; men, 43.6%), and widespread chronic pain (women, 59.7%; men, 40.3%) [32]; in another Australian study, where the rate of chronic pain is higher in adult women (21%) than in men (17%) [33,34]. About half of chronic pain conditions are more prevalent in women [35]. Moreover, women are more likely to experience chronic pain conditions compared with men, usually reporting more pain levels and functional issues than men [36]. Another variable related to pain level was BMI (*p* < 0.001), associated in our sample with an overweight prevalence of 41% and obesity prevalence of 23.3%, with the obesity prevalence being higher the higher the Pain Level: 20.4% (Low Pain) vs. 24.1% (Medium Pain) vs. 29.2% (High Pain), with significant differences between the three groups (*p* < 0.05). This is not surprising given that the relationship between obesity and pain is also clear [37,38]. The rest of the categorial variables also showed dependence relationships with Pain Level: Social Class (*p* < 0.001), Smoking Status (*p* = 0.033), PAF (*p* < 0.001), SPH (*p* < 0.001), 500 mWD (*p* < 0.001), and 12SD (*p* < 0.001).

By checking SHP as a function of PAF in each of the Pain Level groups, it is found that Negative SHP decreases as PAF increases, so inactive people in the three groups are those with the highest Negative SHP prevalence. Several studies have explored the relationships between these variables. One study found that pain was the most important factor in classifying SPH, ahead of others such as sleep, dyspnoea, fatigue, or depression in older people [39]. Another study with adults and older respondents found that a majority who experienced mild and moderate pain considered their health to be good, while those with severe or maximum pain considered their health to be fair [40].

According to the findings on functional mobility difficulties, both in walking 500 m and in going up and down 12 steps, there was a high prevalence of limitations in inactive people at each of the pain levels. Alternatively, expressed in another direction, people with functional mobility limitations are very inactive. When examining the scientific literature, it is found that chronic pain is associated with changes in mobility [41], resulting in a continuum that ranges from altered movements to complete avoidance [42,43]. Another study of 7490 older people assessed associations between self-reported pain and ADL impairment and concluded that pain, especially musculoskeletal and multi-location pain, significantly affects ADLs, which involve functional mobility [44]. Stamm et al. [45] found that older people with musculoskeletal diseases had a higher frequency of ADL problems than people without these diseases. The ADLs with more issues were “doing heavy housework” (43.9%), bending or kneeling (39.3%), walking up and down stairs without a walker (23.1%), and walking 500 m without a walker (22.8%).

Based on the results of the binary multiple logistic regressions performed for SPH taking as reference the highest PAF (Very Frequently), lower PAF levels were associated with higher probability risks of perceiving a Negative SPH. Pain (high, 6.22; medium 2.88) would be the greatest risk factor for having a Negative SPH, but physical inactivity would be the next greatest risk factor, with inactive people being 2.39 (CI95%: 2.12; 2.70) times more likely to have a negative SPH than very active people. Moreover, low PAF was also associated with higher odds of reporting 500 m walking difficulties compared to the higher PAF group (Never (OR: 5.09; CI95%: 4.09; 6.33; *p* < 0.001) and Occasionally (OR:1.76; CI95%: 1.41; 2.20; *p* < 0.001)); and the same applied with the difficulties of walking up or down 12 steps (Never (OR: 3.55; CI95%: 2.98; 4.23; *p* < 0.001) and Occasionally (OR: 1.64; CI95%: 1.37; 1.95; *p* < 0.001)). Physical activity is a non-pharmacological treatment for pain [46], so its reduction may cause a worsening of the pain state. In addition, inactivity is a risk factor for developing or worsening other comorbidities, affecting morbidity and mortality [47] and frailty in the case of older people [48]. O’Neill et al. [48] obtained that older adults who did not meet PA guidelines were at a higher risk of moving from the No Pain class to the High Impact Pain class. Niederstrasser and Attridge [49] performed a cross-sectional and longitudinal study to assess the Associations between pain and PA in older adults; in their cross-sectional analysis, they found that all PA levels were associated with a reduced risk of musculoskeletal pains, while in the longitudinal one, they observed that only high PA was associated with a lower probability of reporting being troubled by musculoskeletal pain ten years later.

### 4.2. Practical Applications, and Future Lines

On the one hand, PA improves health and well-being, and on the other hand, physical inactivity and sedentary behaviour increase the risk of suffering from a variety of diseases that impact people’s quality of life and health [2]. Thus, states and other actors should commit and invest to comply with WHO-recommended guidelines on PA and sedentary behaviour [3], to reduce the burden on health systems as well as to align with the Sustainable Development Goals [50]. Moreover, gender differences, and other sociodemographic variables (age, sex, BMI, social class, civil status and, smoking status) must be attended to, both at the diagnostic and treatment level, considering the importance of awareness raising and training of professionals in this field [51].

### 4.3. Strength and Limitations

A strength of this study is that it was conducted through several surveys over different periods of time, with a large enough sample size of the Spanish population to ensure the reliability of the results. Additionally, the type of analysis performed adds to the robustness of the findings. In our logistic regression analysis, we considered a wide range of potential effect-modifying variables, including age, sex, BMI, civil status, social class, smoking status, pain level, and PAF.

The limitations of this study include the fact that, being a cross-sectional study, it is not possible to establish causal relationships. Moreover, the extent of pain symptoms could not be analysed according to body location or extent, as the survey did not provide this information. Furthermore, PAF was self-reported, unaccompanied by the use of objective devices to measure PA as is already being carried out in other surveys [52]. It would be interesting to carry out research with designs which allow the establishment of causal relationships. Longitudinal studies could help to establish PA’s real impact on people with pain and contribute to further knowledge in this field, including the optimal dose adjustment to achieve benefits in this population [46].

## 5. Conclusions

In the present study, associations were found between higher pain levels and worse SPH, and more functional mobility limitations, in this case, in walking 500 m and up and down 12 steps, among middle-aged and elderly people living in Spain with different pain levels. In all Pain Level groups, a lower PAF was related to a worse SPH and difficulties in functional mobility. Physical inactivity is presented as a risk factor, being the most important after pain (high and moderate), for negative SPH and walking and stair climbing limitations.

These associations underline the importance that PA programmes can play in the improvement of health and functional mobility, as well as other aspects, in people with pain. Future longitudinal studies could clarify the true impact of physical activity on people with pain and help determine the optimal exercise dosage for this population.

## Data Availability

The original contributions presented in the study are included in the article/Supplementary Material; further inquiries can be directed to the corresponding authors.

## Supplementary Materials

The following supporting information can be downloaded at: https://www.mdpi.com/article/10.3390/jfmk9040198/s1, Table S1: Descriptive Analysis. Table S2: SPH according to PAF. Table S3: 500 m Walking Difficulties according to PAF. Table S4: 12 Steps Difficulties according to Physical Activity Frequency. Tables S5–S7: Regression Models.
