# Supplementary material for: Relationship Between Frequency of Physical Activity, Functional Mobility, and Self-Perceived Health in People with Different Levels of Pain: A Cross-Sectional Study
Source: J Funct Morphol Kinesiol. 2024 Oct 21;9(4):198. doi: 10.3390/jfmk9040198 (PMC11503292; doi:10.3390/jfmk9040198)
Supplement: Supplementary file 1 [file jfmk-09-00198-s001.zip › Supplementary Material/Table S1. Descriptive Analysis.pdf]

Table S1. Descriptive Analysis.

	Total n=21,162		Low Pain n=10,220		Medium Pain n=7022		High Pain n=3920					
Variables	Median	IQR	Median	IQR	Median	IQR	Median	IQR	X^2	df	$p^{K\text{-}W}$	V
Age	60	19	58	19	60	19	61	19	-	-	<0.001	-
Sex	n	%	n	%	n	%	n	%			p^{X2}	
Men	8161	38.6	4485a	43.9	2501b	35.6	1175c	30.0	269.9	2	<0.001	0.113
Women	13,001	61.4	5735a	56.1	4521b	64.4	2745c	70.0				
BMI_Group												
Underweight	256	1.3	110a	1.1	89a	1.3	57a	1.5	132.5	6	<0.001	0.057
Normal	6964	34.5	3573a	36.5	2241b	33.5	1150c	31.0				
Overweight	8277	41.0	4106a	41.9	2749a	41.1	1422b	38.3				
Obesity	4697	23.3	1999a	20.4	1612b	24.1	1086c	29.2				
Social_Class												
I	1915	9.3	1124a	11.3	553b	8.1	238c	6.3	190.8	10	<0.001	0.068
II	1471	7.1	779a	7.8	485a	7.1	207b	5.5				
III	3902	19.0	1967a	19.7	1291a	19.0	644b	17.0				
IV	3115	15.1	1494a	15.0	1049a	15.4	572a	15.1				
V	7014	34.1	3189a	32.0	2405b	35.3	1420b	37.4				
VI	3157	15.3	1413a	14.2	1027a	15.1	717b	18.9				
Smoking Status												
Smokers	4392	20.8	2114a	20.7	1450a	20.7	828a	21.1	13.7	6	0.033	0.018
Occasionals	377	1.8	193a	1.9	128a	1.8	56a	1.4				
Ex Smokers	6357	30.1	3164a	31.0	2032b	29.0	1161a,b	18.3				
No Smokers	10,028	47.4	4746a	46.5	3407b	48.6	1875a,b	18.7				
PAF												
Never	8730	41.3	3462a	33.9	3073b	43.8	2195c	56.0	613.1	6	<0.001	0.120
Occasionally	8911	42.1	4768a	46.7	2876b	41.0	1267c	32.3				
Frequently	1678	7.9	917a	9.0	535b	7.6	226c	5.8				
Very Frequently	1843	8.7	1073a	10.5	538b	7.7	232c	5.9				
Self-Perceived Health												
Positive	9674	45.7	6419a	62.8	2506b	35.7	749c	19.1	2606.1	2	<0.001	0.351
Negative	11,488	54.3	3801a	37.2	4516b	64.3	3171c	80.9				
500m Walking Difficulties												
Yes	4660	22.0	1044a	10.2	1790b	25.5	1826c	46.6	2255.8	2	<0.001	0.326
No	16,502	78.0	9176a	89.8	5232b	74.5	2094c	53.4				
Up/Down 12 Steps Difficulties												
Yes	6097	28.8	1581a	15.5	2345b	33.4	2171c	55.4	2308.4	2	<0.001	0.330
No	15,064	71.2	8639a	84.5	4676b	66.6	1749c	44.6				
Some Difficulty to Walking or Steps												
Yes	6464	30.5	1701a	16.6	2514b	35.8	2249c	57.4	2352.3	2	<0.001	0.333
No	14,697	69.5	8519a	83.4	4507b	64.2	1671c	42.6				

n (participants); % (percentage); X^2 (Pearson Chi-Square); df (Degree freedom); $p^{K\text{-}W}$ (p-value from Kruskal-Wallis test); p^{X2} (p-value from Chi-Square test); V (Cramer's V coefficient); abc (Differents letters mean significant differences in proportions between Pain Level Groups with p<0.05 from post hoc pairwise z test for independent proportions).
