# Supplementary material for: Relationship Between Frequency of Physical Activity, Functional Mobility, and Self-Perceived Health in People with Different Levels of Pain: A Cross-Sectional Study
Source: J Funct Morphol Kinesiol. 2024 Oct 21;9(4):198. doi: 10.3390/jfmk9040198 (PMC11503292; doi:10.3390/jfmk9040198)
Supplement: Supplementary file 1 [file jfmk-09-00198-s001.zip › Supplementary Material/Table S2. SPH according to PAF.pdf]

Table S2. Self-Perceived Health according to Physical Activity Frequency in People with Low, Medium and High Pain.

People with Low Pain												
Variables	PAF								X^2	df	p	V
Self-Perceived Health	Never (A)		Occasionally (B)		Frequently (C)		Very Frequently (D)					
	n	%	n	%	n	%	n	%				
Positive	1880	54.3	3011	63.2	695	75.8	833	77.6	274.5	3	<0.001	0.164
Negative	1582	45.7	1757	36.8	222	24.2	240	22.4				
Proportions's differences post hoc												
Positive				A (p<0.001) ***		A (p<0.001) ***<br>B (p<0.001) ***		A (p<0.001) ***<br>B (p<0.001) ***				
Negative		B (p<0.001) ***<br>C (p<0.001) ***<br>D (p<0.001) ***		C (p<0.001) ***<br>D (p<0.001) ***								
**People with Medium Pain**												
Variables	PAF								X^2	df	p	V
Self-Perceived Health	Never (A)		Occasionally (B)		Frequently (C)		Very Frequently (D)					
	n	%	n	%	n	%	n	%				
Positive	857	27.9	1065	37.0	277	51.8	307	57.1	251.1	3	<0.001	0.189
Negative	2216	72.1	1811	63.0	258	48.2	231	42.9				
Proportions's differences post hoc												
Positive				A (p<0.001) ***		A (p<0.001) ***<br>B (p<0.001) ***		A (p<0.001) ***<br>B (p<0.001) ***				
Negative		B (p<0.001) ***<br>C (p<0.001) ***<br>D (p<0.001) ***		C (p<0.001) ***<br>D (p<0.001) ***								
**People with High Pain**												
Variables	PAF								X^2	df	p	V
Self-Perceived Health	Never (A)		Occasionally (B)		Frequently (C)		Very Frequently (D)					
	n	%	n	%	n	%	n	%				
Positive	262	11.9	313	24.7	88	38.9	86	37.1	204.6	3	<0.001	0.228
Negative	1933	88.1	954	75.3	138	61.1	146	62.9				
Proportions's differences post hoc												
Positive				A (p<0.001) ***		A (p<0.001) ***<br>B (p<0.001) ***		A (p<0.001) ***<br>B (p=0.001) **				
Negative		B (p<0.001) ***<br>C (p<0.001) ***<br>D (p<0.001) ***		C (p<0.001) ***<br>D (p=0.001) **								

p (p-value from pairwise z-test for independant proportions); * (p<0.05); ** (p<0.01); *** (p<0.001); X^2 (Chi-Square); df (Degree freedom); V (V's Cramer coefficients).
