# Supplementary material for: Relationship Between Frequency of Physical Activity, Functional Mobility, and Self-Perceived Health in People with Different Levels of Pain: A Cross-Sectional Study
Source: J Funct Morphol Kinesiol. 2024 Oct 21;9(4):198. doi: 10.3390/jfmk9040198 (PMC11503292; doi:10.3390/jfmk9040198)
Supplement: Supplementary file 1 [file jfmk-09-00198-s001.zip › Supplementary Material/Table S3. 500m Walking Difficulties according to PAF.pdf]

Table S3. 500m Walking Difficulties according to Physical Activity Frequency in People with Low, Medium and High Pain.

People with Low Pain

Variables	PAF								X^2	df	p	V
500m Walking Difficulties	Never (A)		Occasionally (B)		Frequently (C)		Very Frequently (D)					
	n	%	n	%	n	%	n	%				
No	2805	82.2	4428	93.0	894	97.5	1049	97.9	424.0	3	<0.001	0.204
Yes	609	17.8	332	7.0	23	2.5	22	2.1				
Proportions's differences post hoc												
No				A (p<0.001) ***		A (p<0.001) ***<br>B (p<0.001) ***		A (p<0.001) ***<br>B (p<0.001) ***				
Yes		B (p<0.001) ***<br>C (p<0.001) ***<br>D (p<0.001) ***		C (p<0.001) ***<br>D (p<0.001) ***								

People with Medium Pain

Variables	PAF								X^2	df	p	V
500m Walking Difficulties	Never (A)		Occasionally (B)		Frequently (C)		Very Frequently (D)					
	n	%	n	%	n	%	n	%				
No	1896	64.3	2353	82.2	484	90.8	499	92.8	428.7	3	<0.001	0.250
Yes	1054	35.7	511	17.8	49	9.2	39	7.2				
Proportions's differences post hoc												
No				A (p<0.001) ***		A (p<0.001) ***<br>B (p<0.001) ***		A (p<0.001) ***<br>B (p<0.001) ***				
Yes		B (p<0.001) ***<br>C (p<0.001) ***<br>D (p<0.001) ***		C (p<0.001) ***<br>D (p<0.001) ***								

People with High Pain

Variables	PAF								X^2	df	p	V
500m Walking Difficulties	Never (A)		Occasionally (B)		Frequently (C)		Very Frequently (D)					
	n	%	n	%	n	%	n	%				
No	844	43.2	873	69.7	190	84.1	187	81.7	360.0	3	<0.001	0.314
Yes	1111	56.8	379	30.3	36	15.9	42	18.3				
Proportions's differences post hoc												
No				A (p<0.001) ***		A (p<0.001) ***<br>B (p<0.001) ***		A (p<0.001) ***<br>B (p=0.001) **				
Yes		B (p<0.001) ***<br>C (p<0.001) ***<br>D (p<0.001) ***		C (p<0.001) ***<br>D (p=0.001) **								

p (p-value from pairwise z-test for independant proportions); * (p<0.05); ** (p<0.01); *** (p<0.001); X^2 (Chi-Square); df (Degree freedom); V (V's Cramer coefficients).
