# Supplementary material for: Relationship Between Frequency of Physical Activity, Functional Mobility, and Self-Perceived Health in People with Different Levels of Pain: A Cross-Sectional Study
Source: J Funct Morphol Kinesiol. 2024 Oct 21;9(4):198. doi: 10.3390/jfmk9040198 (PMC11503292; doi:10.3390/jfmk9040198)
Supplement: Supplementary file 1 [file jfmk-09-00198-s001.zip › Supplementary Material/Table S4. 12 Steps Difficulties according to Physical Activity Frequency.pdf]

Table S4. Difficulty climbing up and down 12 or more steps according to Physical Activity Frequency in People with Low, Medium and High Pain.

People with Low Pain

Variables	PAF								X^2	df	p	V
12 Steps Difficulties	Never (A)		Occasionally (B)		Frequently (C)		Very Frequently (D)					
	n	%	n	%	n	%	n	%				
No	2618	76.8	4145	87.2	857	93.5	1019	95.1	336.6	3	<0.001	0.182
Yes	789	23.2	607	12.8	60	6.5	52	4.9				
Proportions's differences post hoc												
No				A (p<0.001) ***		A (p<0.001) ***<br>B (p<0.001) ***		A (p<0.001) ***<br>B (p<0.001) ***				
Yes		B (p<0.001) ***<br>C (p<0.001) ***<br>D (p<0.001) ***		C (p<0.001) ***<br>D (p<0.001) ***								

People with Medium Pain

Variables	PAF								X^2	df	p	V
12 Steps Difficulties	Never (A)		Occasionally (B)		Frequently (C)		Very Frequently (D)					
	n	%	n	%	n	%	n	%				
No	1691	57.7	2064	72.1	461	86.3	460	85.5	324.7	3	<0.001	0.217
Yes	1241	42.3	798	27.9	73	13.7	78	14.5				
Proportions's differences post hoc												
No				A (p<0.001) ***		A (p<0.001) ***<br>B (p<0.001) ***		A (p<0.001) ***<br>B (p<0.001) ***				
Yes		B (p<0.001) ***<br>C (p<0.001) ***<br>D (p<0.001) ***		C (p<0.001) ***<br>D (p<0.001) ***								

People with High Pain

Variables	PAF								X^2	df	p	V
12 Steps Difficulties	Never (A)		Occasionally (B)		Frequently (C)		Very Frequently (D)					
	n	%	n	%	n	%	n	%				
No	683	35.8	722	58.0	176	78.2	168	74.3	308.6	3	<0.001	0.293
Yes	1225	64.2	522	42.0	49	21.8	58	25.7				
Proportions's differences post hoc												
No				A (p<0.001) ***		A (p<0.001) ***<br>B (p<0.001) ***		A (p<0.001) ***<br>B (p<0.001) ***				
Yes		B (p<0.001) ***<br>C (p<0.001) ***<br>D (p<0.001) ***		C (p<0.001) ***<br>D (p<0.001) ***								

p (p-value from pairwise z-test for independant proportions); * (p<0.05); ** (p<0.01); *** (p<0.001); X^2 (Chi-Square); df (Degree freedom); V (V's Cramer coefficients).
