# Supplementary material for: Relationship Between Frequency of Physical Activity, Functional Mobility, and Self-Perceived Health in People with Different Levels of Pain: A Cross-Sectional Study
Source: J Funct Morphol Kinesiol. 2024 Oct 21;9(4):198. doi: 10.3390/jfmk9040198 (PMC11503292; doi:10.3390/jfmk9040198)
Supplement: Supplementary file 1 [file jfmk-09-00198-s001.zip › Supplementary Material/Tables S5-S7. Regression Models.pdf]

Table S5. Multivariate binary logistic regression analysis including Self-Perceived Health as the dependant variable.

	β	OR	C.I. (95%)	p
Age	0.041	1.04	(1.04; 1.04)	<0.001***
BMI (Normal)		Ref.		
Underweight	0.167	1.18	(0.88; 1.58)	0.258
Overweight	0.074	1.08	(1.00; 1.16)	0.045*
Obesity	0.268	1.31	(1.20; 1.43)	<0.001***
PAF (Very frequently)		Ref.		
Never	0.872	2.39	(2.12; 2.70)	<0.001***
Occasionally	0.459	1.58	(1.41; 1.78)	<0.001***
Frequently	0.155	1.17	(1.00; 1.36)	0.048*
Social Class (I)		Ref.		
II	0.154	1.17	(1.00; 1.36)	0.053
III	0.302	1.35	(1.19; 1.54)	<0.001***
IV	0.587	1.80	(1.58; 2.05)	<0.001***
V	0.719	2.05	(1.82; 2.31)	<0.001***
VI	0.846	2.33	(2.04; 2.67)	<0.001***
Pain Level (Low)		Ref.		
Medium	1.059	2.88	(2.69; 3.09)	<0.001***
High	1.828	6.22	(5.65; 6.84)	<0.001***
Civil Status (Single)		Ref.		
Married	-0.143	0.87	(0.79; 0.95)	0.002**
Divorced	-0.187	0.83	(0.73; 0.94)	0.005**
Legally Separated	-0.035	0.97	(0.80; 1.16)	0.711
Widowed	0.037	1.04	(0.90; 1.20)	0.609
Constant	-3.985	0.02		

β (Beta); OR (Odds ratio); Ref. (Reference); C.I. (Confidence interval); p (p-value); * (p-value<0.05); ** (p-value<0.01); *** (p-value<0.001).

Table S6. Multivariate binary logistic regression analysis including Limitations on walking 500m as the dependant variable.

	β	OR	C.I. (95%)		p
Age	0.067	1.07	(1.07;	1.07)	<0.001***
Sex (Men)		Ref.			
Women	-0.131	0.88	(0.81;	0.96)	0.003**
BMI (Normal)		Ref.			
Underweight	0.552	1.74	(1.20;	2.52)	0.004**
Overweight	0.179	1.20	(1.08;	1.33)	0.001**
Obesity	0.643	1.90	(1.71;	2.12)	<0.001***
PAF (Very frequently)		Ref.			
Never	1.626	5.09	(4.09;	6.33)	<0.001***
Occasionally	0.568	1.76	(1.41;	2.20)	<0.001***
Frequently	0.157	1.17	(0.87;	1.57)	0.300
Social Class (I)		Ref.			
II	0.240	1.27	(0.98;	1.65)	0.073
III	0.565	1.76	(1.43;	2.17)	<0.001***
IV	0.507	1.66	(1.34;	2.05)	<0.001***
V	0.790	2.20	(1.81;	2.68)	<0.001***
VI	0.974	2.65	(2.15;	3.26)	<0.001***
Pain Level (Low)		Ref.			
Medium	0.999	2.72	(2.46;	3.00)	<0.001***
High	1.871	6.49	(5.83;	7.24)	<0.001***
Constant	-8.247	0.00			

β (Beta); OR (Odds ratio); Ref. (Reference); C.I. (Confidence interval); p (p-value); * (p-value<0.05); ** (p-value<0.01); *** (p-value<0.001).

Table S7. Multivariate binary logistic regression analysis including Limitations to up or down 12 Steps Stairs as the dependant variable.

	β	OR	C.I. (95%)	p
Age	0.071	1.07	(1.07; 1.08)	<0.001***
Sex (Men)		Ref.		
Women	0.081	1.08	(1.00; 1.17)	0.043*
BMI (Normal)				
Underweight	0.537	1.71	(1.21; 2.41)	0.002**
Overweight	0.185	1.20	(1.10; 1.32)	<0.001***
Obesity	0.779	2.18	(1.97; 2.41)	<0.001***
PAF (Very frequently)				
Never	1.266	3.55	(2.98; 4.23)	<0.001***
Occasionally	0.492	1.64	(1.37; 1.95)	<0.001***
Frequently	0.089	1.09	(0.86; 1.39)	0.465
Social Class (I)				
II	0.211	1.23	(0.99; 1.54)	0.063
III	0.459	1.58	(1.32; 1.89)	<0.001***
IV	0.494	1.64	(1.37; 1.97)	<0.001***
V	0.708	2.03	(1.72; 2.40)	<0.001***
VI	0.855	2.35	(1.96; 2.81)	<0.001***
Pain Level (Low)				
Medium	0.932	2.54	(2.33; 2.77)	<0.001***
High	1.779	5.92	(5.36; 6.55)	<0.001***
Constant	-7.797	0.00		

β (Beta); OR (Odds ratio); Ref. (Reference); C.I. (Confidence interval); p (p-value); * (p-value<0.05); ** (p-value<0.01); *** (p-value<0.001).
